# Richmond Academy of Medicine Meeting

**Published:** 1895-06

**Authors:** 


### Society Notes.

### Richmond, Va., Academy of Medicine Meeting, April 23, 1895.

The president, Wm. S. Gordon, in the chair. Dr. J. Allison Hodges, leader on the subject selected for the evening's discussion, read a paper on Some of the Diagnostic Nervous Manifestations of Syphilis.

The nervous symptoms are more manifest in proportion to the absence of cutaneous symptoms. All the nervous symptoms are not always dependent on syphilis; a number of nervous diseases have their origin in this malady. In diagnosing the disease, the medication method is unreliable, some other affections being improved by it. The nervous symptoms may be developed in each stage of the disease, but it is in the tertiary stage principally that the gravest lesions of the nervous system appear, and, since it is especially in those cases where the ordinary secondary manifestations were wanting that we are to expect these complications, it is important for the physician, in making such a diagnosis, to be prepared to recognize the first danger signals that may be manifested. The primary stage has no prominent nervous symptoms, those present being referable rather to the concomitant anæmia than to the action of the specific poison. The secondary stage presents more marked evidences of implication of the nervous system—various neuralgias, dyspepsia of nervous origin, cardiac palpitations and meningitis, cerebral or spinal, being characteristically present. The tertiary stage gives evidence of numberless shades and varieties of nervous affections, and in this period of the disease, the nervous symptoms manifested are due solely to the influence of the specific virus circulating in the blood and irritating the delicate nervous structures.

The symptoms produced may be those due to an inflammation or degeneration of the nerve centres themselves, or to the effects produced by pressure upon the nerve centres or trunks by product of this same form of inflammation located in contiguous structures—the symptoms all showing lesions either of the intracranial organs or of the spinal cord, less frequently of the spinal nerves.

The diagnostic symptoms detailed, are also diagnostic of other diseases. It is by association that we determine the disease, as locality, etc. There are periodic occipital headaches in nearly every case, absent in the forenoon, returning most frequently at night and becoming worse. I have never found any tenderness on pressure. The diagnostic nervous manifestations of syphilis are: 1. Headache, which disappears if paralysis occur. 2. Insomnia, nearly always associated with headache, and disappearing with the appearance of convulsions or paralysis. It differs from the insomnia of neuræsthenia and melancholia in that it occurs in the early night, the victim arising in the morning ready for his daily labor. 3. Vertigo, occurring usually with the headache. It may be transient, but becomes worse as the disease progresses. 4. Convulsions. In the adult they are not preceded by convulsions in youth. 5. Tremor, present in one-half of cases. It occurs most often, in the order named, in the hands, tongue and over the whole body, and is accompanied by headache. If it occur in a limb, it is the precursor of paralysis of the limb. 6. Hemiplegia. 7. Erratic distribution of paralysis, as aphasia with or without hemiplegia; ptosis; insanity or epilepsy with paralysis of one arm or leg. It is suggested that ptosis occurring suddenly points nearly always to syphilis. 8. The use of electricity to determine central or peripheral lesion. 9. The presence of great physical weakness and mental dullness. This is one of the most valuable of the nervous manifestations, being out of proportion to the seeming condition of the patient. 10. History of the case. In women the history of many abortions in suuccession would point to syphilis.

In the treatment of syphilis, the iodide should be given in sufficiently potential doses in Carlsbad or other waters. The cases I report show how easy it is to overlook the disease in the tertiary stage, when the first and second were not noticeable. In conclusion let me say we could often abort syphilis by studying the nervous system and giving treatment in time.

